# Passive Performance Evaluation and Validation of a Viscous Impeller Pump for Subpulmonary Fontan Circulatory Support

**DOI:** 10.21203/rs.3.rs-2584661/v1

**Published:** 2023-03-01

**Authors:** Weiguang Yang, Timothy A. Conover, Richard S. Figliola, Guruprasad A. Giridharan, Alison L. Marsden, Mark D. Rodefeld

**Affiliations:** 1 Departments of Pediatrics (Cardiology), Stanford University; 2 Departments of Mechanical Engineering, Clemson University; 3 Department of Bioengineering, University of Louisville; 4 Department of Bioengineering, Stanford University; 5 Section of Cardiothoracic Surgery, Indiana University School of Medicine

## Abstract

Patients with single ventricle defects undergoing the Fontan procedure eventually face Fontan failure. Long-term cavopulmonary assist devices using rotary pump technologies are currently being developed as a subpulmonary power source to prevent and treat Fontan failure. Low hydraulic resistance is a critical safety requirement in the event of pump failure (0 RPM) as a modest 2 mmHg cavopulmonary pressure drop can compromise patient hemodynamics. The goal of this study is therefore to assess the passive performance for a viscous impeller pump (VIP) we are developing for Fontan patients, and validate flow simulations against in-vitro data. Two different blade heights (1.09 mm vs 1.62 mm) and a blank housing model were tested using a mock circulatory loop (MCL) with cardiac output ranging from 3 to 11 L/min. Three-dimensional flow simulations were performed and compared against MCL data. In-silico and MCL results demonstrated a clinically insignificant pressure drop of < 2 mmHg at a cardiac output of 7 L/min for both blade heights. There was good agreement between simulation and MCL results for pressure loss (mean difference −0.23 mmHg 95% CI [0.24 −0.71]). Compared to the blank housing model, low wall shear stress area and oscillatory shear index on the pump surface were low, and mean washout times were within 2 seconds. This study demonstrated the low resistance characteristic of current VIP designs in the failed condition that results in clinically acceptable minimal pressure loss with low risk of thrombosis.

## Introduction

The Fontan procedure is the third and final stage of the typical palliative surgical sequence for children born with single ventricle heart defects. In the Fontan procedure, the inferior vena cava (IVC) is routed to the pulmonary artery (PA) via an extracardiac expanded polytetrafluoroethylene (ePTFE) graft or intra-atrial lateral tunnel baffle. Systemic venous return is completely diverted to the PA forming a total cavopulmonary connection (TCPC) after Fontan completion. Despite greatly reduced early death following the Fontan procedure, long term morbidity and mortality remain unsatisfactory due to inefficiencies inherent in a univentricular Fontan circulation which stem from the lack of a subpulmonary pump. Recent studies have shown that Fontan failure and late complications are responsible for 30 out of 40 deaths, with freedom from late Fontan complications of only 53% and 31% at 5 and 20 years, respectively^1^. Overall 30-year survival after Fontan repair is only 43% for a single center cohort of 1052 patients^2^.

Fontan physiology produces near-normal oxygen saturation at the expense of elevated systemic venous pressure, relative pulmonary arterial hypotension and reduced cardiac output compared to a normal biventricular physiology; these factors form the basis of the so-called “Fontan paradox.” Chronic and progressive elevation in systemic venous pressure, liver, intestinal, and lymphatic disorders, subnormal cardiac output, and elevated pulmonary capillary wedge pressure and systemic vascular resistance all contribute to the syndromic constellation of Fontan-associated disease^3^. Interestingly, however, in a large portion of patients with failing Fontan physiology, ventricular systolic function remains preserved^4^. This suggests that Fontan failure is not secondary to primary pump failure, but is rather secondary to chronic preload deprivation which has been postulated to lead to pathologic ventricular remodeling and diastolic dysfunction that contribute to progression of late Fontan failure^3,5^. In other words, Fontan failure is not a failure of the single ventricle per se, but is rather a failure to fill the ventricle. Given this, we postulated that the “Fontan paradox” can be reversed by adding a mechanical cavopulmonary assist device that is capable of creating a subpulmonary pressure rise of 5-7 mm Hg from the vena cavae to the PAs to restore the equivalent of a normal 2-ventricle circulation^6,7^.

Although ventricular assist devices (VADs) have been used for failing Fontan patients as a bridge to heart transplant^8-10^, existing mechanical circulatory assist devices are mainly designed for the high-pressure systemic circulation and are ill-suited for use in the Fontan circulation, which requires only a modest pressure rise. It is however critical to assure long-term patency of the Fontan circuit in the event of pump failure. Rodefeld et al.^11^ proposed a viscous impeller pump (VIP) design inspired by the von Karman viscous pump which allows for 4 direction flow augmentation using a single pump. Several groups have followed with proof of concept for similar Fontan pumps, based on shrouded impeller designs^12,13^. In addition, axial flow pump designs have been pursued for supporting the Fontan circulation as well^6,14-17^.

Our team is further refining the VIP design toward the goal of clinical translation. The pump utilizes an outrunner motor configuration in which an outer biconical rotating component consists of permanent magnets and 4 hydraulic blades on each side, with stationary motor windings located centrally. The pump is suspended by struts in the midst of the cavopulmonary junction to continuously augment blood flow from the SVC and IVC to the PAs through a widely patent primary flow path. A thin film secondary flow path allows a portion of flow to provide bearing lubrication and heat dissipation. Preliminary data show that a prototype pump can achieve a pressure head up to 14 mmHg at 5600 revolutions per minute (RPM) with no measurable gradient to passive Fontan flow at 0 RPM^18^.

The requirement for long-term patency of the Fontan circuit in the event of pump failure poses a design challenge as one must balance the tradeoff between competing objectives for active and passive flow. On one hand, the device should provide the desired pressure head rise with best possible efficiency; on the other hand, it should provide minimal internal resistance for a broad range of performance across various metabolic states, have low propensity for suction and cavitation, and most importantly not obstruct the Fontan circuit in the event of rotational failure. Note that a modest 2 mmHg pressure drop in Fontan passive flow can compromise pulmonary afterload and cardiac output requiring intervention^19^. In prior work, we have applied the surrogate management framework (SMF) to optimize the palliative surgeries for single ventricle defects^20-23^. However, before design optimization can be carried out for VIPs, it is necessary to validate our baseline simulations against in-vitro experimental data under passive flow conditions. Thus, the goal of this study is to compare the pressure gradient across the pump at 0 RPM predicted by computational fluid dynamics (CFD) to experimental measurements in a mock circulatory loop (MCL) for a wide range of flow conditions and to characterize the passive hemodynamic performance of current VIP designs. The active hemodynamic performance under rotating conditions is presented in a separate study^24^.

## Methods

### Mock Circulatory Loop

**Figure 1.**
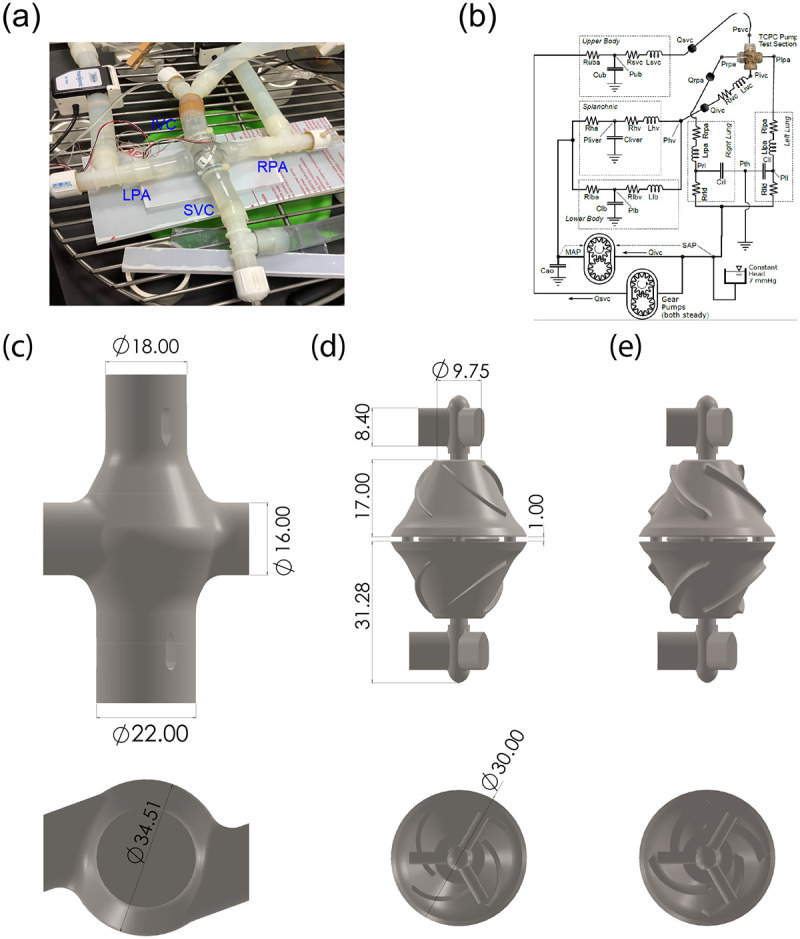
MCL layout and VIP prototype design. (a) MCL Test section. Several Y and T shaped tube fittings are used at the inlets and outlets of the test section for catheter access. The internal diameter for inflow and outflow tubing is 1.91 cm. (b) A diagram of the mock circulatory system. Physical lumped parameter models are coupled to the pump forming a closed loop circulation. (c) VIP housing. (d) The baseline VIP design employs 4 impellers with a height of 1.09 mm for each inlet. (e) The modified design increases the blade height to 1.62 mm with edge fillets. Dimensions are in mm.

The MCL is an in-vitro test platform consisting of hydraulic and mechanical components to simulate blood circulation under healthy and pathological conditions. Previously MCLs have been extensively used to characterize hemodynamics in congenital heart diseases and VADs^25-30^. The VIP was installed inside a transparent pump housing with 2 inlets and 2 outlets connected to the pipes that represent the venae cavae and the PAs, respectively (Figures 1a-b). To test the influence of geometry, two impeller designs and a blank housing model were compared (Figures 1c-e). The baseline design contained blades with a height of 1.09 mm while in the modified design, the blade height was increased to 1.62 mm and edge fillets were used to smooth sharp edges. The impeller and rotor hub surface were 3D printed by Pro Jet 700HD (3D Systems, Rock Hill, SC, United States) using a translucent polycarbonate like material (Accura ClearVue) with a smooth and glossy finish and mounted to the rotor. The printer is capable of reproducing features down to 0.05 mm. As a control, the blank housing model had the identical geometry except with no pump and struts inside.

In the upstream and downstream portions of the test section, five blocks of physical lumped parameter models (LPMs) were used to model the circulation in the lower body, upper body, splanchnic organs and two lungs (Figure 1b). The resistance elements in the MCL were implemented by adjustable ball valves and laminar flow elements in which bundles of small elastic tubes with diameters ranging from 0.75 to 3 mm were packed together. The systemic compliance elements in MCL were implemented by “windkessels” which are cylinders (D=102 mm) containing specific volumes of trapped air^28^. The two pulmonary compliance elements employ the hydrostatic principle, each having a specific area of free surface in a vertical prism to create pressure proportionate to stored volume.

Two computer-controlled gear pumps were used to independently drive upper and lower body flow, and therefore SVC and IVC flow, with a split ratio of 60/40 (IVC/SVC) in this study. Together, they generated 5 cardiac output (CO) values of 3.0, 5.0, 7.0, 9.0 and 11.0 L/min. Atrial pressure of 7.5 mmHg was maintained by a constant head tank. A glycerol/water mixture with a weight ratio of 40/60 (mass ratio) is used as a blood analog fluid with a density of 1.06 g/cm3 and a viscosity of 0.035 Poise. The changes in fluid temperature were within 1 degree Celsius. Based on a diameter of 1.91 cm for the inflow tubing and IVC flow, the Reynolds number was 674, 1015, 1433, 1839 and 2250 for CO = 3.0, 5.0,7.0, 9.0 and 11.0 L/min, respectively. The pulmonary impedance networks caused the RPA and LPA outflow pressures to vary according to 6.0+0.74×CO and 6.0+0.77×CO, respectively. Four liquid-filled pressure catheters were placed at 4 cm from the pump housing center. Volumetric flow rates were measured by ultrasonic flow sensors installed at the upstream and downstream of each inlet and outlet, respectively. An in-house Labview program was used for data acquisition. Pressure and flow signals were filtered by 4th-order and 1st-order filters, respectively, before sampling. Pressure and flow drifts were kept within 0.01 L/min and 0.05 mmHg at the initial and final all-stop measurements.

### Passive Flow Simulations

The test section and its adjacent tubing were created using SolidWorks 2019 (Dassault Systèmes SolidWorks Corporation, Waltham, MA, United States). The solid file was discretized into linear tetrahedral elements using the MeshSim software library (Simmetrix Inc., Clifton, NY, United States). The general isotropic mesh size and the tubing surface mesh size were set to 0.2 and 0.09 cm respectively. To represent the complex geometry of the pump, the surface mesh size for impellers and struts was reduced to 0.03 cm and 0.06 respectively. To improve the resolution near the wall, we used a boundary layer mesh in which the mesh size at the n-th innermost layer is reduced to $\frac{1}{2^{n}}h$, where *h* is the general isotropic mesh size. To reduce computational cost, n was set to 3 and 1 for the central test section and peripheral tubing, respectively. The resulting mesh consists of 7.9 and 3 million tetrahedral elements for a model with a VIP and a blank housing model respectively. The mean tetrahedron aspect ratio, minimum dihedral angle and shape of the baseline VIP model are 2.14, 39.55 and 0.69, respectively. A mesh sensitivity study was performed by comparing the pressure loss at the highest cardiac output for the baseline VIP design on coarsened and refined meshes. The differences in pressure loss were within 3%. Additional details are presented in the supplementary materials.

The stabilized finite element Navier-Stokes solver svSolver available open source via the SimVascular project (https://simvascular.github.io/)^31-33^ was employed to simulate the flow testing. The source code for the flow solver is available on Github (https://github.com/SimVascular/svSolver). The weak form for incompressible Newtonian flow with no-slip rigid walls is as follows, Find {v,p}∈S for any {w,q}∈W such that

(1) $$B_{G}(w,q;v,p)=\int_{\Omega }w\cdot (\rho v_{,t}+\rho v\cdot \nabla v-f)d\Omega +\int_{\Omega }\nabla w:(-pI+\tau )d\Omega \;\;-\int_{\Gamma_{h}}\{w\cdot h\}d\Gamma +\int_{\Omega }q\nabla \cdot vd\Omega =0,$$

where ***v*** and *p* are velocity and pressure in trial solutions space *S*, respectively, ***w*** and *q* are weighting functions for the momentum and continuity equations in weighting space *W* respectively, ***f*** and ***τ*** are body force and viscous stress tensor respectively, Ω represents the fluid domain, and Γ_*h*_ represents Neumann boundaries with traction forces ***h*** prescribed on vessel outlet faces. Note that Equation 1 was augmented to include residual based stabilization terms^34,35^ in the solver to overcome the instability due to advection-dominated flow and the equal order interpolation of velocity and pressure. A second-order generalized-*α* method was employed for time integration and a Newton-Raphson method was employed to solve the nonlinear equation at the correction step^36^. In addition, outlet back flow stabilization^37^ and resistance based preconditioning^38,39^ techniques were employed in the solver.

We imposed the flow rates measured in the MCL testing by prescribing parabolic velocity profiles at the inlets. At the outlets, we used a resistance boundary condition^40^ in which the traction ***h*** in Equation 1 is related to downstream resistance *R* and outflow *Q* by $h=-RQn=-R\int_{\Gamma_{h}}u\cdot nd\Gamma n$. The resistance values for the RPA and LPA outlets were chosen to be 204.51 and 222.49 dyn·s/cm^5^ to achieve the measured flow split (R52/L48) and PA pressures for the blank housing model at CO=11 L/min and kept unchanged for other cases.

Steady flow simulations were performed for 5 seconds with a time step of 0.001 s. State files were saved every 0.02 second. A python script using the paraview.simple module was created to extract and average the pressures data on cut planes at 4 cm away from the origin from *t* = 2s to *t* = 5s for calculation. The risk of thrombosis is evaluated using common shear-stress-based endpoints. Low wall shear stress (WSS) and oscillatory shear index (OSI) are associated with flow recirculation that increases the risk of thrombosis. The time-averaged wall shear stress (TAWSS) and OSI are defined as follows,

(2) $$\tau =\sigma \cdot n-(\sigma \cdot n\cdot n)n,\;\;TAWSS=|\frac{\int_{0}^{T}\tau dt}{T}|,\;\;OSI=\frac{1}{2}\left(1-\frac{|\int_{0}^{T}\tau dt|}{\int_{0}^{T}|\tau |dt}\right),$$

where ***n*** is the unit normal vector and ***σ*** is the stress tensor. Regions with TAWSS < 5 dyn/cm^2^ are empirically defined as low WSS regions based on previous studies^41-43^.

To characterize the regions of flow stasis, we placed a 3.5×3.5×3.5 cm cube of virtual dye with uniform concentration in the center of the pump and quantified the required time for the dye to be washed out. Similarly, a stabilized finite element solver was employed to solve the following weak form of the advection-diffusion equation^44^.

Find ϕ∈S for any q∈W such that

(3) $$\int_{\Omega }q\cdot (\phi_{,t}+\rho v\cdot \nabla \phi )d\Omega +\int_{\Omega }\nabla q\cdot (\kappa +\kappa_{DC}\nabla \phi )d\Omega +\Sigma_{e}\int_{\Omega_{e}}\nabla q\cdot v\tau_{m}ℒ(\phi ,v)d\Omega =0,\;\;\tau_{m}(x,t)=\frac{1}{\sqrt{4∕dt^{2}}+v^{T}\vec{\xi }v+3(\kappa +\kappa_{DC}{)}^{2}\xi :\xi }\;\;\kappa_{DC}=\frac{|ℒ|}{2\sqrt{\nabla^{T}\phi \xi \nabla \phi }},$$

where ***ϕ*** is a scalar field representing the dye concentration, ***v*** is the velocity field given by the solution of Equation 1, *κ* and *κ*_*DC*_are the diffusion coefficient and the discontinuity capturing diffusion coefficient, $\tau_{m}$ is the stabilization parameter and ℒ is the advection-diffusion operator $ℒ\frac{\partial \phi }{\partial t}+v\cdot \nabla \phi -\nabla \cdot \kappa \nabla \phi$.

The initial scalar field ϕ(x) was set to be 1 in the region of interest and 0 elsewhere. The boundary value at inlets were set to be 0. The washout time T(x) is defined as the minimum time needed for ***ϕ*** to decrease to 1% of its maximum value so that:

(4) $$T(x)=\int_{t_{max}}^{t_{1\%}}dt,$$

where *t*_*max*_ is the time when ***ϕ*** starts to decay from the maximum value and *t*_1%_ is the time when ***ϕ*** reaches 1% of its maximum value at ***x***.

## Results

**Figure 2.**
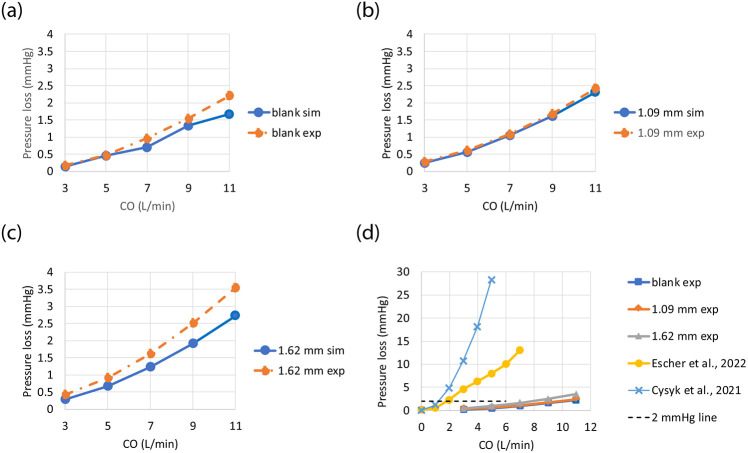
Pressure drops across (a) a blank housing and a static pump with (b) 1.09 mm blades and (c) 1.62 mm blades under 5 cardiac output values measured by simulations and MCS. In (d), in-vitro pressure loss data for 0 RPM are compared between the present study and previous studies by Escher et al.^45^ and Cysyk et al.^12^ in which two shrouded Fontan pumps were studied. The dashed line represents a threshold value of 2 mmHg for interventions in Fontan patients.

Figures 2a-c shows the differences in pressure loss between CFD and MCL at 3, 5, 7, 9 and 11 L/min. The largest difference from the experimental data (−0.8 mmHg) was found in the 1.62 mm model at the highest flow rate of 11 L/min. Experimental data shows that the difference in pressure loss due to the changes in blade height (1.09 mm vs 1.62mm) was up to 1.1 mmHg at 11 L/min while simulations predicted a difference of 0.5 mmHg under the same condition. Compared to the blank housing model, the increased pressure loss due to the presence of a failed VIP is at most 0.7 mmHg at CO=7 L/min. Although these pressure gradients may at first appear to be trivial, in the Fontan circuit, an obstruction of 2 mmHg or greater is clinically significant. The results from the present design are thus in a range that is thought to be safe and clinically tolerable. Figure 2d further compares the in-vitro pressure loss at 0 RPM between the VIPs and two shrouded pumps proposed by Graneger et al.^13,45^ and Cysyk et al.^12^. Compared to the VIP used in the present study, the pressure loss across a failed shrouded pump rises markedly with increasing flow.

**Figure 3.**
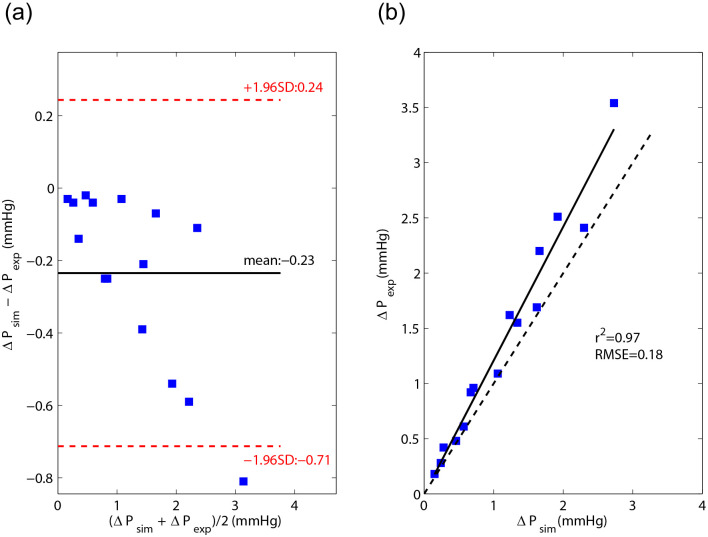
Bland-Altman (a) and correlation (b) plots for simulation and MCS derived pressure drop data.

Differences between simulation and MCL data increased with increasing cardiac output (Figure 3). Overall, there was good agreement and correlation between simulation and MCL results, though simulations tended to slightly under-predict the pressure drop compared to MCL.

Power loss caused by the pump and housing was compared to the values reported in previous Fontan simulation studies^46-50^. The static pump resulted in a similar or smaller power loss compared to conventional and Y-shaped Fontan models (Supplementary Figure S3).

**Figure 4.**
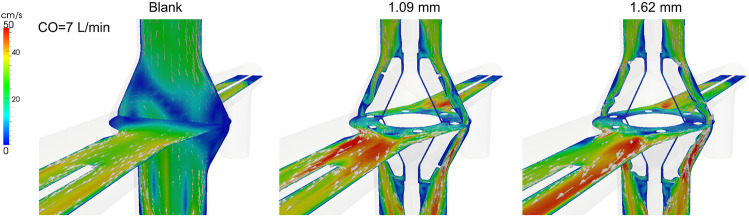
Mean velocity magnitude and vectors projected onto two central cut planes at 7 L/min.

Representative velocity fields in three models are visualized in Figure 4. A recirculation zone is visible in the blank housing. The presence of a static VIP eliminated that recirculation zone and increased flow velocity though local flow separation remained and can be seen near the blades and the equator where the upper and lower rotors are connected.

**Figure 5.**
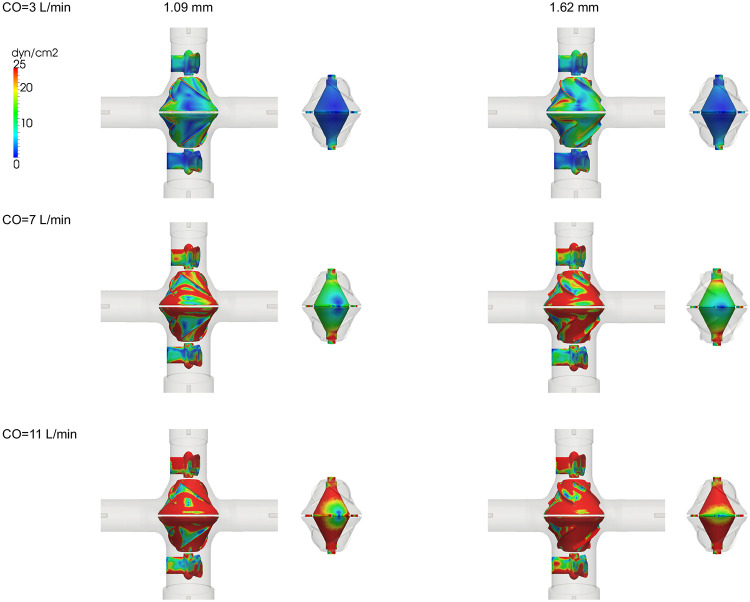
Time averaged wall shear stress (TAWSS) distribution on the rotor, strut and secondary flow surfaces for a 1.09 mm blade design and a filleted 1.62 mm blade design.

**Figure 6.**
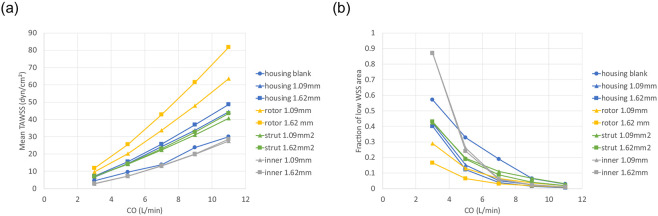
(a) Mean wall shear stress (WSS) and (b) area fraction of low WSS ≤ 5 dyn/cm^2^ for the housing, rotor, strut and inner secondary flow surfaces.

Figure 5 show the TAWSS distribution for the pump surface. Mean TAWSS values and the areas of low TAWSS (< 5 dyn/cm^2^) for different parts of the pump are shown in Figure 6. The blank housing model resulted in a lower WSS level and an increased region of low WSS on the housing surface compared to the models with a VIP inside (Supplementary Figure S4). Compared to a conventional extracardiac Fontan graft (D=20 mm)^46^, there is a larger chamber at the junction in the blank housing model (Dmax=34 mm) creating more flow recirculation. Due to a reduced cross-sectional area for flow passage, mean TAWSS for both VIPs was greater than those of the blank housing model and increased with increasing blade height. Since flow in the secondary flow path was less than 1% of the total inflow, a lower WSS level is expected there.

OSI on the rotor and strut surfaces was nearly zero for CO ≤ 5 L/min. The size of the region with non-zero OSI increased with increasing CO due to flow separation past the rotor blades and struts (Supplementary Figure S5 ). In contrast, the flow in the secondary path was nearly unidirectional regardless of cardiac output.

**Figure 7.**
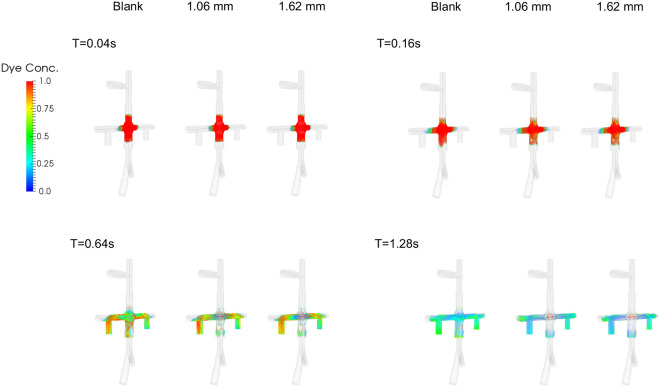
Dye concentration at *T* =0.04, 0.16, 0.64*s* for a blank housing, 1.09 mm blades and 1.62 mm blades at CO=3 L/min. Virtual dyes with ***ϕ*** = 1 were placed in the center of the pump at *T* = 0 and advected with flow.

**Figure 8.**
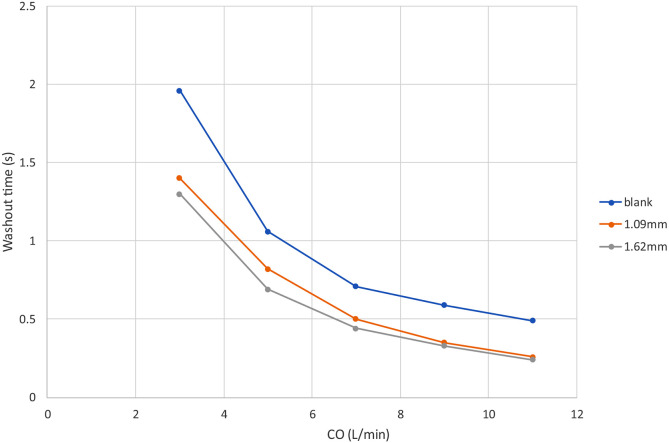
Mean washout time for dyes initially released in the pump under different flow conditions.

Figures 7 shows the dye concentration at *T* =0.04, 0.16, 0.64s for a × 3.5 × 3.5 cm cube of virtual dyes initially released at the center of the pump for CO=3 L/min. Residual dyes are visible near the wall or in the secondary flow path only for the VIP models while the blank housing model still show a large amount of dyes in the center after 0.64s (Figure 7). With increased cardiac output, after 0.64s, most dye is concentrated in the secondary flow path only for the VIP models while residual dye remains visible near the housing wall for the blank housing model (Supplementary Figure S6). Figure 8 shows the mean washout time in the region of interest under all flow conditions. We found that it took more time to wash out the dyes in the blank housing model than in the VIP models where regions with high washout time were located in the secondary path and junctions only.

## Discussion

Unlike the existing mechanical circulatory devices placed in parallel to the native circulation, the VIP is permanently implanted in-series in the lowest pressure venous segment of the human circulation. A minimal risk of flow obstruction in the event of mechanical failure is a critical safety requirement as the in-series placement creates complete circulatory dependency on flow passing through the device. Novel to blood pump design, a pump intended for Fontan circulatory support must thus be equally optimized to maximize required Fontan circulatory support while avoiding obstruction potential in its functional (rotating) state as well as its failed (non-rotating) state. Balancing the requirement for optimal pump function (hydraulic efficiency, head rise, low power requirement) with the need for minimal risk in a failed state (passive flow optimization, thrombogenicity risk mitigation) are competing objectives that are diametrically opposed, and present a unique challenge. Patients with Fontan circulation survive for decades after Fontan procedure. Current rotary blood pump technologies, while approved for destination therapy, are limited to 5-10 years of use. Thus, the challenge of having a failure tolerant, low pump resistance design that can provide adequate support when functional is critical to safely translate cavopulmonary assist to clinical practice.

In this study, we validated the simulation predicted pressure loss for two VIP prototypes and a blank housing model under passive flow conditions (i.e. a non-rotating or “failed” pump at 0 RPM) against MCL testing data. Overall, there was excellent agreement for cardiac output values of 3, 5 and 7 L/min. The differences between simulations and MCL increased up to 0.8 mmHg for high cardiac outputs of 9 and 11 L/min due to increased flow disturbances. Despite steady inflow conditions, the flow field became unsteady with increasing cardiac output as flow separation and vortex shedding were created by the interactions between flow and internal structures including the pump blades, hub surface and struts. Factors that may have contributed to slightly higher discrepancies at higher cardiac output values include the catheter placement and geometric discrepancies between pump/tubing and discretized computational domains.

Both MCL and numerical modeling confirmed a low resistance design which produces clinically insignificant pressure drops. In clinical practice, catheter or surgical intervention for conduit stenosis is indicated for a pressure gradient exceeding 2 mmHg in the Fontan circuit under resting conditions^51,52^. Since the pressure gradient for the VIPs tested in this study is less than 2 mmHg at CO= 7 L/min, the hydraulic resistance of a static VIP is in a clinically insignificant range. Our design intent is that at 0 RPM, the biconical design splits SVC and IVC flow without significantly increasing resistance. This failure mode is comparable to previous Fontan passive flow modifications such as the OptiFlo and Y-graft designs^46,47,53-55^. In contrast, shrouded Fontan pumps proposed by others have reported a clinically untenable gradient of 13 mmHg for a flow rate of 7 L/min^45^ and 18 mmHg for a flow rate of 4L/min^12^. Fontan circuit gradients in this range would be clinically destabilizing and likely necessitate emergency surgical intervention for salvage. From a safety perspective, we believe that intrinsic low hydraulic resistance is essential for a long-term Fontan pump. In other words, a Fontan patient should never be dependent upon pump flow, or upon the pump remaining operational for survival.

The design of a long term cavopulmonary assist device is subject to unique objective functions and constraints that may be in conflict with each other. Careful consideration must be made as placing emphasis on different aspects of pump performance may lead to diametrically opposed directions in the VIP design. With increased geometric complexity, we need to better understand the relationships between design parameters and quantities of interest. Design refinements could be better informed by the use of sensitivity analysis and shape optimization techniques in future studies^20,23,56-59^.

In addition to low hydraulic resistance, risks of thrombosis also play a role in the long-term passive safety of VIPs. Despite a lack of accurate and reliable models for predicting thrombosis formation, three shear-stress-based endpoints were used to characterize flow stasis in the region of interest. Regions of low WSS in the VIP models were localized showing a smaller area fraction of low WSS compared to the blank housing model despite a larger blood contacting area in the pump. As compared to the 1.09 mm design, the increased mean WSS on the rotor surface (+27% at 7 L/min) and reduced low WSS area fraction (−3% at 7 L/min) can be attributed to the use of edge fillets that improved flow separation despite a larger pressure drop in the 1.62 mm design. Overall mean TAWSS in the VIPs was in the physiologic range^43^ and comparable to the mean TAWSS in the extra cardiac Fontan graft^46,54,55^ for CO=3 and 5 L/min.

Similarly, dye washout time results agreed with the shear-based endpoints. Dye in the secondary flow path was trapped at 3 L/min taking more than 5 seconds to be washed out and making the secondary flow path prone to blood clots when the pump is stopped. However, the passive performance is unlikely to be affected by an obstructed secondary flow path given that < 1% of the venous return passes through the secondary flow path. Other flow stasis regions under low cardiac outputs include the junction between the housing and outlets, and the base of the conical rotor hub surface suggesting a local shape modification could be made to improve flow separation and recirculation. Overall, our simulation results examining surrogates for thrombotic potential indicate a low risk for thrombosis with the VIP design.

## Limitations

A major limitation of this study is the use of steady inflow conditions. Although unsteady vortex shedding and fluctuations developed with increasing cardiac output, the instantaneous flow field remains different from the natural condition with respiration-dependent pulsatile flow. For time-averaged quantities such as pressure drops and TAWSS, we expect to find similar trends among the three models tested in this study under pulsatile flow conditions. The increased pressure drop that would occur at the peak inspiratory flow would likely be similar to the results under higher steady flow conditions (9 and 11 L/min).

The passive performance for the VIP in a patient specific model was not evaluated in this study. Unlike the test section in which circular pipes were arranged on the same plane, there is a large variation in patient specific PAs. In future studies, patient specific models coupled with a closed loop lumped parameter network^60-62^ will be studied.

In addition, the flow split for the IVC/SVC was fixed at 60/40 and symmetric pulmonary flow distribution was only considered in this study. Previous studies showed that IVC flow fraction is correlated with age and body surface area^63,64^, and unilateral pulmonary arteriovenous malformation can lead to skewed pulmonary flow distribution^41^. Thus, the impacts of uncertainties in inflow and outflows splits on pump active and passive performance should be studied to account for younger patients and patients with uncommon flow conditions in future studies.

A threshold value used for assessing low WSS has not been validated and the risks of thrombosis were compared relative to the blank housing model. It is well known that an oversized extracardiac Fontan graft is prone to thrombosis despite lower hydraulic resistance^65^. The blank housing model with a bulging chamber at the Fontan junction creates extra space for mixing and recirculation resembling an oversized graft. However, these findings need to be validated compared to future in-vitro thrombosis studies, and furthermore, future hypothetical studies on the impact of clots virtually added to the pump surface will help to establish the performance envelop.

The fluid is assumed to be Newtonian. Although this is generally a valid approximation for flow in large vessels with large shear rates^66^, non-Newtonian effects related to thrombogenesis may become important in flow recirculation where shear rates are low. Predictions for the locations where thrombi develop will be validated against future in-vitro and animal in-vivo studies.

## Conclusions

Both MCL and simulation results demonstrated that the latest VIP design created clinically insignificant pressure drops at 0 RPM under most flow conditions in Fontan patients. We observed good agreement between simulations and MCL results. Low WSS area and dye washout time in the pumps with both blade heights were smaller than those of a blank housing model. With an ≤ 0.5 mmHg increase in the pressure gradient at 0 RPM for CO=3-7 L/min and greater hydraulic heads under working conditions, the 1.62 mm design with edge fillets is preferred to the 1.09 mm design. These findings will serve as a foundation for future design optimization to enhance the long-term passive safety features of cavopulmonary assist devices.

## Data Availability

The data generated during the current study are available from the corresponding author on reasonable request. The source code for the flow solver is available on Github (https://github.com/SimVascular/svSolver).
